# Hypothyroidism in Severe Burn Patients Associated with Increased Risk of Musculoskeletal Complications and Decreased Risk of Mortality

**DOI:** 10.1093/jbcr/iraf090

**Published:** 2025-05-22

**Authors:** Isabel Obias, Dalton Amador, Sudhanvan Iyer, Amina El Ayadi, George Golovko, Steven E Wolf, Juquan Song

**Affiliations:** John Sealy School of Medicine, University of Texas Medical Branch, Galveston, TX, United States; John Sealy School of Medicine, University of Texas Medical Branch, Galveston, TX, United States; John Sealy School of Medicine, University of Texas Medical Branch, Galveston, TX, United States; Department of Surgery, University of Texas Medical Branch, Galveston, TX, United States; Department of Pharmacology, University of Texas Medical Branch, Galveston, TX, United States; Department of Surgery, University of Texas Medical Branch, Galveston, TX, United States; Department of Surgery, University of Texas Medical Branch, Galveston, TX, United States

**Keywords:** hypothyroidism, burns, contractures

## Abstract

Severe burn patients are prone to developing various musculoskeletal, dermatological, and cardiovascular complications, and hypothyroidism is associated with derangements of these same systems. This study aims to explore the association between hypothyroidism and the emergence of these complications in severe burn patients. Patients with severe burns, defined as those encompassing ≥20% total body surface area, were identified in the TriNetX US Collaborative Network and were categorized into 2 groups based on a history of hypothyroidism. Risk ratios (RRs) and differences of contractures, undergoing graft procedures, graft complications, amputations, and various infections and cardiovascular complications were generated using the database’s measure of association analysis. The hypothyroidism cohort showed a greater risk of developing contractures (RR, 1.667; 95% confidence interval [CI], [1.207, 2.302]; *P* = .002), skin infections (RR, 1.885; CI, [1.192, 2.980]; *P* = .006), and urinary tract infections (RR, 1.950; CI, [1.155, 3.292]; *P* = .011) within 6 months of insult. The hypothyroidism cohort showed a decreased risk of mortality (RR, 0.688; CI, [0.516, 0.915]; *P* < .010), undergoing graft procedures (RR, 0.647; CI, [0.518, 0.807]; *P* < .001), and vasopressor use (RR, 0.806; CI, [0.667, 0.974]; *P* = .025) in this same time period. In conclusion, hypothyroidism in severe burn patients is associated with an increased risk of developing contractures and skin and urinary tract infections and a decreased risk of mortality, undergoing graft procedures, and vasopressor use.

## INTRODUCTION

Hypothyroidism is defined as sustained decreased production of thyroid hormones, including triiodothyronine (T3) and thyroxine (T4). Thyroid hormones have an intrinsic role in homeostasis and the metabolic rate and function of every organ system. Untreated hypothyroidism leads to a wide array of systemic and metabolic ailments and classically presents as fatigue, cold intolerance, weight gain, constipation, and dry skin, among other manifestations.^1^ Importantly, because of the disruptive effect of hypothyroidism on normal physiology of the cardiovascular, musculoskeletal, and integumentary systems, patients therewith often experience delayed wound healing and are susceptible to various secondary complications to trauma.^2^

Depending on the severity of injury, burns lead to homeostatic disruption of many organ systems. Those who suffer from severe burns, traditionally defined as those encompassing at least 20% of the total body surface area (TBSA), often experience systemic and metabolic derangements immediately after and in the months following the initial insult.^3^ Skin, muscle, and connective tissue are commonly affected, and the musculoskeletal and integumentary function of injured areas may be impaired and recovery prolonged. Limbs may be rendered unsalvageable with especially severe injury, requiring amputation.^4^ Repairing this damage is energetically costly, necessitating a prolonged hypermetabolic state that results in systemic and collateral damage to other organ systems; this hypermetabolic response is associated with increased mortality.^5^

Little data exists on the effects of preexisting hypothyroidism in those who experience severe burns. Because of the essential role of active thyroid hormones in homeostasis and wound healing, we wonder whether they may be at increased risk of developing infections related to delayed wound healing and various musculoskeletal and integumentary complications. Additionally, we wondered if hypothyroidism counteracts severe burns’ hypermetabolic response, leading to decreased mortality. The purpose of this study is to investigate whether preexisting hypothyroidism as a risk factor in burns for complications related to wound healing and recovery, and its effect on overall mortality in this population.

## METHODS

We utilized information gathered from the TriNetX US Collaborative Network, a federated network comprising electronic medical record (EMR) data sourced from 60 healthcare organizations (HCOs). These organizations consist predominantly of academic medical centers and encompass various entities such as academic-owned or affiliated clinics, primary and satellite hospitals, physician practices, and specialized treatment centers. The participating HCOs refresh data approximately once per week. The TriNetX platform is neutral regarding EMR systems and offers de-identified data, including demographics, diagnoses, procedures, medications, laboratory values, and genomics, covering an estimated 104 million patients. The de-identification process adheres to the guidelines of the General Data Protection Regulation and the Health Insurance Portability and Accountability Act, specifically outlined in Section §164.514(b)^1^ of the HIPAA Privacy Rule. This compliance renders the TriNetX platform exempt from informed consent (UTMB IRB No. 20-0085).

**Figure 1.**
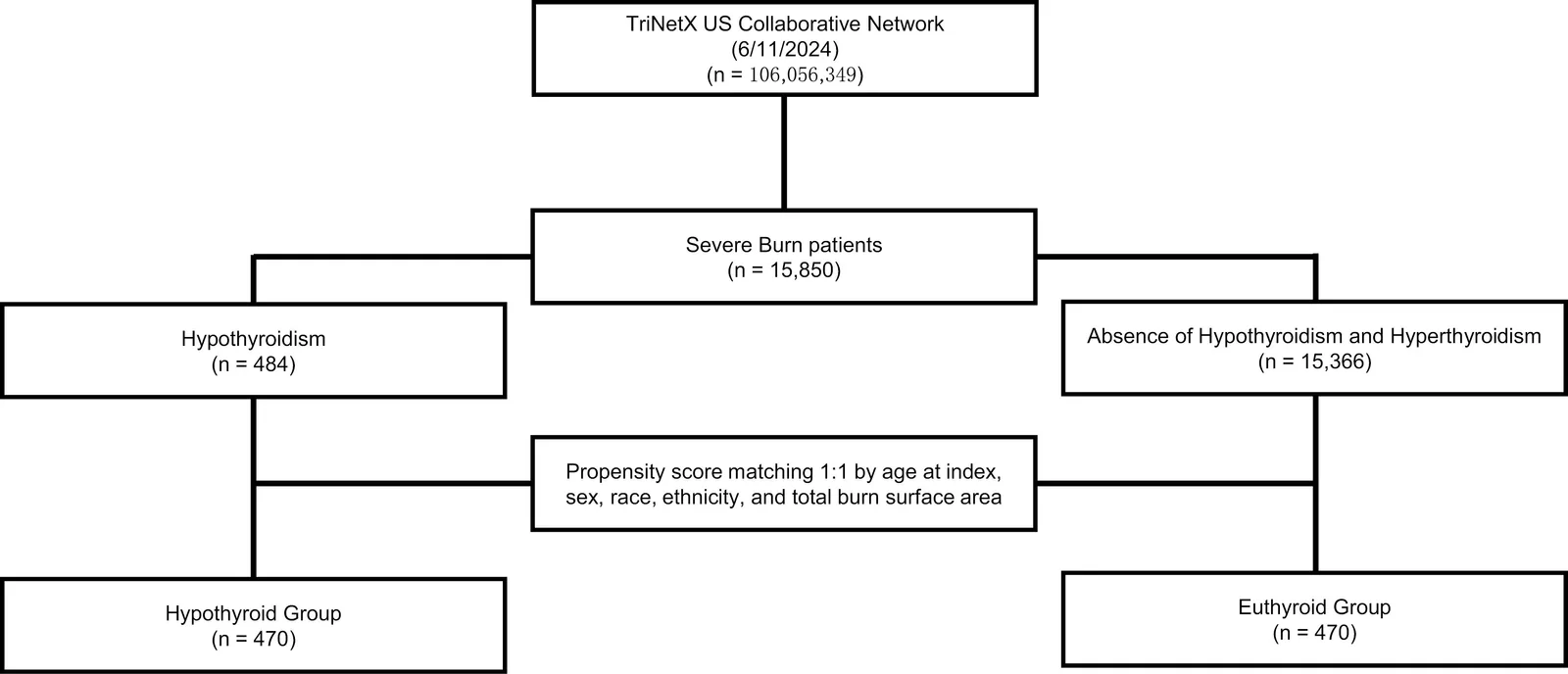
Flow Chart for Cohort Selection.

The current study included EMR data from the TriNetX US Collaborative Network on severe burns collected from June 11, 2003 to June 11, 2023. A flowchart of the cohort construction is shown in **Figure 1**. Males and females of all ages who were diagnosed with burns equating to 20% TBSA or greater were identified for the study (ICD-10 codes T31.2-31.9). To ensure that our cohort consisted of patients with acute burns rather than those returning for reconstructive procedures, we included only the first recorded instance of burn diagnosis and examined associated surgical interventions occurring within 1 week of initial presentation. Patients with isolated reconstructive procedures, without concurrent acute burn diagnosis, were excluded from the analysis. Additionally, the dataset was filtered to include only major HCOs, thereby minimizing the likelihood of capturing outpatient follow-up visits rather than acute burn cases. These were stratified into 2 groups based on any preexisting history of hypothyroidism. Hypothyroidism was defined using the ICD-10 code E03.9. The groups were propensity score matched 1:1 for age, sex, race, ethnicity, and TBSA burned. This 1:1 matching approach was implemented as a standard by the TriNetX platform, which employs nearest neighbor matching without replacement and a default caliper of 0.10 standard deviations. This method is designed to optimize the balance between cohorts while minimizing selection bias. Currently, the TriNetX interface does not support alternative matching ratios.

A descriptive analysis was done on the 2 cohorts to compare baseline demographic information. A simple *t*-test was used to compare the average age at index and the standard deviation of the 2 cohorts. Minor discrepancies in sample sizes post-matching resulted from missing data in certain demographic variables. Patients lacking complete demographic information were excluded from pairwise analyses, leading to slight variations in reported totals. $\chi {}^{2}$ or Fisher’s exact tests were used to compare categorical data such as sex, race, and ethnicity.

The groups were subsequently compared within 6 months postburn. Given that many patients do not remain hospitalized for 6 months, readmissions were identified based on unique patient identifiers and subsequent inpatient encounters occurring within 30 days of discharge. The following common postburn infectious complications were compared: sepsis (A41.89, A41.9), skin infections (L00-L08), urinary tract infections (UTI) (N39.0), and pneumonia (J18). N39.0 (UTI, site not specified) was included as it encompasses a broad range of UTIs documented in various clinical settings. While more specific codes exist for conditions such as cystitis, we aimed to capture the most inclusive representation of UTIs in this patient population. Similarly, E03.9 (unspecified hypothyroidism) was used to include the broadest range of patients with chronic hypothyroidism, while excluding congenital and secondary forms of hypothyroidism that may involve distinct pathophysiological mechanisms. Cardiovascular and other hemodynamic complications, including myocardial infarction (I21), hypotension (I95), and volume depletion (E86), were also compared. Additionally, the following musculocutaneous complications were compared: contractures (M62.4, M24.5, L90.5, M67, M62.89) and amputations (see **Supplementary Material**). Finally, we compared various interventions, reconstructive operations, and concomitant complications common with severe burns between the 2 groups, including autograft and skin substitute graft procedures (CPT 1003391 and 1020888, respectively), graft complications (T86.821, T86.82), intubation (CPT 31500), and the use of pressors (VA AU100). For all outcomes of interest, risk ratios (RR), risk differences, and 95% confidence intervals were calculated within the TriNetX system. *P* values < .05 were considered significant.

## RESULTS

A total of 15 850 patients were identified in the TriNetX system as severe burn patients. 484 patients were sorted into the group with a history of hypothyroidism (“hypothyroid group”). 15 366 patients were sorted into the group with no history of hypothyroidism or hyperthyroidism (“euthyroid group”). After propensity score matching 1:1 by age at index, sex, race, ethnicity, and total burn surface area, 470 severe burn patients were selected for the hypothyroidism group and the euthyroid group.

A comparison of demographic information between the hypothyroid and euthyroid groups before and after matching is displayed in **Table 1**. The average age at index of the hypothyroidism group was 59 years old, which was significantly older than the overall euthyroid group at 39.2 years old. The groups also differ in sex distribution, with the hypothyroid group being predominantly female (56.9%) and the euthyroid group overall being predominantly male (69.1%). The hypothyroid group was predominately white (73.2%), followed by African Americans (10.1%) and Latinos (5.7%). Similarly, the overall euthyroid group was mostly white (57.4%) but consisted of more minorities, with 16% African American and 11.2% Latino.

**Table 1.** Demographics of Hypothyroid and Euthyroid Group Before and After Matching.

	Before propensity matching			After propensity matching		
Characteristics	Hypothyroid (*n* = 484)	Euthyroid (*n* = 15 366)	*P* value	Hypothyroid (*n* = 470)	Euthyroid (*n* = 470)	*P* value
Age at index						
Mean ± SD	59.0 ± 16.5	39.2 ± 22.5	<.001	58.9 ± 16.4	59.9 ± 16.9	.375
Sex						
Male	185 (39.1)	9,154 (69.1)	<.001	185 (39.4)	192 (40.9)	.641
Female	269 (56.9)	3,872 (29.2)	<.001	268 (57)	260 (55.3)	.599
Ethnicity						
Not Hispanic or Latino	374 (79.1)	8,889 (67.1)	<.001	373 (79.4)	379 (80.6)	.625
Unknown ethnicity	72 (15.2)	2,885 (21.8)	.001	70 (14.9)	74 (15.7)	.717
Hispanic or Latino	27 (5.7)	1,482 (11.2)	<.001	27 (5.7)	17 (3.6)	.123
Race						
White	346 (73.2)	7,614 (57.4)	<.001	345 (73.4)	342 (72.8)	.825
Black or African American	48 (10.1)	2,122 (16)	.001	48 (10.2)	52 (11.1)	.672
Unknown race	54 (11.4)	2,625 (19.8)	<.001	54 (11.5)	57 (12.1)	.762
American Indian or Alaska Native	10 (2.1)	71 (0.5)	<.001	10 (2.1)	0 (0)	.001
Asian	10 (2.1)	254 (1.9)	.758	10 (2.1)	10 (2.1)	1
Native Hawaiian or Other Pacific Islander	10 (2.1)	51 (0.4)	<.001	10 (2.1)	10 (2.5)	1
Other race	10 (2.1)	254 (1.9)	.758	10 (2.1)	10 (2.1)	1

Before matching, the hypothyroid group demonstrated an increased risk of sepsis, skin infections, UTI, hypotension, volume depletion, myocardial infarction, graft failure, and mortality, and a reduced number of graft procedures at 1 month. However, we found no significant difference in rates of sepsis, hypotension, volume depletion, myocardial infarction, or graft complications between the 2 groups after matching (**Table 2**). After matching, mortality within 6 months of burn injury was significantly lower in the hypothyroid group. The hypothyroid group was also associated with a significantly increased risk of contractures, skin infections, and UTIs, and a reduced risk of vasopressor use and undergoing graft procedures during this same time period.

**Table 2.** Outcomes Risk Ratio (RR) and 95% Confidence Interval (CI) After Matching Comparing the Hypothyroid and Euthyroid Group.

Outcomes	Hypothyroid (*n* = 470)	Euthyroid (*n* = 470)	RR	95% CI	*P* value
Deceased	14.00%	20.40%	0.688	(0.516, 0.915)	.01^a^
Contracture	18.10%	10.90%	1.667	(1.207, 2.302)	.002^a^
Amputation	2.10%	3.20%	0.667	(0.303, 1.469)	.311
Graft procedures	20.60%	31.90%	0.647	(0.518, 0.807)	<.001^a^
Graft complications	5.50%	4.70%	1.182	(0.680, 2.055)	.553
Skin infection	10.40%	5.50%	1.885	(1.192, 2.980)	.006^a^
Sepsis	7.70%	5.50%	1.385	(0.850, 2.256)	.189
UTI	8.60%	4.20%	1.95	(1.155, 3.292)	.011^a^
Pneumonia	8.10%	10.60%	0.76	(0.508, 1.136)	.179
Myocardial infarction	2.80%	2.30%	1.182	(0.535, 2.611)	.689
Hypotension	10.00%	9.10%	1.093	(0.738, 1.620)	.657
Volume depletion	4.70%	4.30%	1.1	(0.609, 1.988)	.752
Vasopressors	28.30%	35.10%	0.806	(0.667, 0.974)	.025^a^
Intubation	7.70%	10.40%	0.735	(0.487, 1.108)	.139

^a^ *P* < .05.

Abbreviation: UTI, urinary tract infection.

## DISCUSSION

In this study, 470 severe burn patients with a history of hypothyroidism were matched and compared to 470 severe burn patients with a history of normal thyroid function. In the 6 months after burn injury, the hypothyroid group was associated with an increased risk of developing contractures, skin infections, and UTIs. In that same time frame, the hypothyroid group showed a decreased risk of mortality, undergoing graft procedures, and vasopressor use. One potential explanation for the reduced vasopressor use in hypothyroid patients is the attenuated adrenergic response associated with hypothyroidism. Since thyroid hormones regulate cardiovascular sensitivity to catecholamines, patients with hypothyroidism may exhibit lower baseline cardiac output and reduced vasopressor requirements, which may partially counteract the hypermetabolic response seen in severe burn injuries.

Thyroid hormones have an essential role in the physiology and function of the cardiovascular system, bone, muscle, and skin, though the exact mechanisms of such are complex and not completely understood.^5,6^ Specifically, these hormones have nuclear receptors and thus influence the transcription and repression of various genes by binding to thyroid hormone response elements.^7^ Thus, over-expression and under-expression of tissue-specific genes—normally existing in a precisely calibrated and regulated transcriptome—inevitably have effects on overall function, classically defined as hypothyroid myopathy and myxedema.^8^ Additionally, primary hypothyroidism is associated with a wide range of other rheumatic symptoms and specific conditions, including generalized muscle and joint weakness and stiffness, myalgias, arthralgias, adhesive capsulitis, carpal tunnel syndrome, trigger finger, Dupuytren’s contracture, tarsal tunnel syndrome, and osteoarthritis.^9^ Less studied is the role thyroid hormones play in the process of wound healing after trauma, and how their deficiency may affect specific elements thereof.

Wound healing relies on the ad hoc expression of a panoply of genes controlling a variety of processes, including collagen proliferation, epithelization, and angiogenesis. It is perhaps unsurprising that hypothyroidism has a significant and negative effect on its overall function.^2^ Most immediately, hypothyroidism results in delayed wound healing, with decreased collagen production often cited as a primary cause.^10^ Likewise, it suggested that a hypothyroid state results in reduced scar tissue formation, though data, especially in humans, is sparse.^11^ This seemingly runs in contrast to our findings that patients with hypothyroidism have an increased risk of developing contractures and early development of hypertrophic scarring after burn injury, both of which result from excessive collagen proliferation. Perhaps this is related to decreased muscular function and the dynamic between active range of motion activities and the development of contractures. An additional consideration is the role of burn severity in these outcomes. Patients with smaller TBSA burns or more superficial injuries may not require grafting but remain at risk for hypertrophic scarring and contractures. Conversely, deeper burns necessitate grafting but may not show the same propensity for contracture formation. While our study did not specifically control for burn depth, future research should incorporate this variable to better elucidate the relationship between thyroid function and burn recovery. Contractures and hypertrophic scarring frequently develop in patients who experience severe burn injury.^12–14^ Contractures, defined as restricted range of motion in any joint secondary to excessive scar formation, occur in up to one-third of these patients; surgical correction is often necessary, though recurrence is common. Hypertrophic scarring, in which excessive scar tissue develops over the skin, is even more common, occurring in up to 70% of these patients. The respective pathophysiologies of contractures and hypertrophic scarring are similar, involving disordered fibroblasts that become overly active secondary to extensive injury. It is notable that hypothyroidism is also associated with disordered fibroblasts, which classically deposit glycosaminoglycan and cause myxedema.^15^

One of the more striking results of our study is that those with hypothyroidism experience decreased mortality at both 1 month and 1 year after injury. Severe burns result in a compensatory hypermetabolic state that lasts months or years after injury.^16,17^ Though necessary to repair the widespread tissue destruction and organ dysfunction severe burns cause, this hypermetabolic state causes its own suite of comorbid sequelae secondary to profound elevations in catecholamines, cortisol, and inflammatory cells and cytokines, including impaired wound healing, systemic catabolism, and increased susceptibility to infection. Interventions that ameliorate the hypermetabolic response, such as beta blockers, ketoconazole, and insulin, are associated with decreased morbidity and mortality.^16^ Another factor that may influence these outcomes is euthyroid sick syndrome (ESS). ESS is characterized by transient reductions in free T3 and T4 without an elevation in thyroid-stimulating hormone, representing an adaptive response to systemic stress rather than true hypothyroidism. While our study focused on preexisting hypothyroidism, it is possible that some patients classified as hypothyroid were experiencing ESS secondary to their burn injuries. This distinction is important, as transient thyroid dysfunction in acute illness may have different implications compared to chronic hypothyroidism. Future studies incorporating serial thyroid function testing could help clarify this relationship.

Hypothyroidism is characterized by a hypometabolic state that may counteract the hypermetabolic response of severe burns, though data on the subject is lacking. Sener et al. found that burn-induced rats who received propylthiouracil, an anti-thyroid pharmacological agent, demonstrated reduced hepatic and gastrointestinal injury compared to the control, which they attributed to attenuated oxidative stress that occurs secondary to burn injury.^18^ Comparable findings in humans are absent in the literature to our knowledge, though it has been shown that a history of hyperthyroidism is associated with an increased risk of morbidity and mortality in severe burn patients.^19^ Future studies may investigate the use of hyperthyroid medications, such as PTU and methimazole, in severe burn patients and its effect on morbidity and mortality, in a similar vein to the current use of beta blockers, ketoconazole, and insulin. Contrastingly, very low free T3 hormone levels characteristic of ESS is associated with increased mortality in this patient population.^20^

It should be noted that these findings rely on data sourced from the TriNetX system, which relies on participating HCOs and administrative coding. Therefore, real-world data will be incomplete in some areas with holes in coding, particularly for some potentially confounding variables. Therefore, we did not control for various diseases and conditions in which hypothyroidism may be secondary, such as diabetes mellitus, lupus, and cancer-related treatment, which may have confounded our results. Additionally, due to the de-identified nature of the TriNetX database, medication administration records, including thyroid hormone replacement therapy, were not available. As a result, we were unable to differentiate between treated and untreated hypothyroidism in our cohort. Since thyroid hormone therapy may mitigate some of the systemic effects of hypothyroidism, this distinction could have important implications for patient outcomes. Future studies should examine whether treatment status influences morbidity and mortality in severe burn patients. In conclusion, we have shown that a history of hypothyroidism is associated with increased risk of contractures, skin infections, and UTI in patients within 6 months of severe burn injury. Additionally, we have shown that hypothyroidism is associated with decreased mortality, vasopressor use, and graft procedures in this same population. Though our findings suggest possible changes to standard clinical management in these patients, future research on these subjects is needed.

## CONCLUSION

Our retrospective propensity-matched cohort study found that severe burn patients with a history of hypothyroidism were associated with a decreased risk of mortality, vasopressor use, and undergoing graft procedures within 6 months after burn injury. Additionally, these same patients were found to be associated with an increased risk of developing contractures, skin infections, and UTIs in this same time period.

## Supplementary Material

iraf090_suppl_Supplementary_Materials

## Data Availability

All relevant data are available within the presented manuscript. Any material and information generated during the study will be available for sharing with other researchers under appropriate institutional agreements. Any inquiries should be directed to the corresponding author.
