# Supplementary material for: Hypothyroidism in Severe Burn Patients Associated with Increased Risk of Musculoskeletal Complications and Decreased Risk of Mortality
Source: J Burn Care Res. 2025 May 22;46(6):1326–31. doi: 10.1093/jbcr/iraf090 (PMC12822486; doi:10.1093/jbcr/iraf090)
Supplement: Supplementary file 1 [file iraf090_suppl_supplementary_materials.docx]

**Supplementary Material**

Amputation CPT codes

1005524

1005529

1004834

28820

1004836

1005298

1014590

26951

27880

28810

1005146

1014580

27590

28825

1005525

28805

27886

26952

26910

27596

27884
